# Populism and foreign policy: a research agenda (Introduction)

**DOI:** 10.1057/s41295-021-00255-4

**Published:** 2021-09-14

**Authors:** Sandra Destradi, David Cadier, Johannes Plagemann

**Affiliations:** 1 Department of Political Science, University of Freiburg, Rempartstr. 15, 79085 Freiburg, Germany; 2 Department of International Relations and International Organization, University of Groningen, PO box 716, 9700 AS Groningen, The Netherlands; 3 Institute for Asian Studies, German Institute for Global and Area Studies (GIGA), Rothenbaumchaussee 32, 20148 Hamburg, Germany

## Introduction

The worldwide rise of populist governments represents one of the most crucial political developments of recent years. In Europe in particular, a range of populist parties and leaders have been voted into power and have formed (or joined) governments over the past decade—this is true, for instance, in Austria, Estonia, Finland, Greece, Hungary, Italy or Poland. As populist actors leave the opposition to seize the reins of executive power, they have entirely new possibilities to directly shape not only domestic policies, but also their countries’ foreign policy and European politics more generally. This could have important repercussions on the European integration project, on relations among European member states and with external powers such as Russia and China, on EU policies in areas such as migration or support to democratization, and on international norms and organizations more generally.

Yet, surprisingly, how and to what extent populist government formation (or participation) concretely influences foreign policy has not been studied systematically so far. Particularly after the election of Donald Trump in the United States, this question has been the object of many (often sensationalist) comments in the media and in policy debates. Also, the literature on the international dimensions of populism has been growing rapidly (see below), but methodical, theory-driven and evidence-based analyses of the impact of populism on foreign policy are still scarce. While the literature on the domestic causes and manifestations of populism has been thriving for decades in the field of Comparative Politics (see, among many others, Meny and Surel 2002; Rooduijn 2018; Norris and Inglehart 2019; Taggart and Kaltwasser 2016), the scholarship exploring the implications of the rise of populism for international and regional politics is, indeed, still in its infancy.

The interdisciplinary dialogue between, on the one hand, Comparative Politics (CP) and Political Theory and, on the other hand, International Relations (IR) and Foreign Policy Analysis (FPA) remains insufficiently developed and structured on these issues. For long, the CP literature studying the influence of populism on policy-making has failed to consider the foreign policy domain while, by contrast, migration or more recently health have received attention (Schain 2006; Zaslove 2012; van Ostaijen and Scholten 2014; Albertazzi and McDonnell 2015; Falkenbach and Greer 2018; Lasco 2020). On its part, the IR literature had largely ignored the populist phenomenon altogether, including the liberal and constructivist works studying, respectively, domestic preference formation and ideational factors (Doyle 2016; Flockhart 2016).

The nascent and burgeoning scholarship on the nexus between populism and foreign policy has begun to address some of these divides and limitations. Yet, several gaps remain. First, in addition to being insufficiently interdisciplinary, this scholarship has often lacked eclecticism in its conceptualization of populism (Chryssogelos 2017, 14). The great majority of studies have relied on the so-called ideational approach (Balfour et al. 2016; Verbeek and Zaslove 2015; Destradi and Plagemann 2019; Wehner and Thies 2020), a few have mobilized the discursive approach (Wojczewski 2019b; Cadier and Szulecki 2020; Jenne 2021), but none have, to our knowledge, relied on the stylistic or politico-strategic approaches or have sought to combine several of these conceptual lenses (see below for an overview of these approaches). Second, most studies have taken on to map, characterize, and generalize from the foreign policy preferences of populist actors (Mead 2011; Verbeek and Zaslove 2017; Chryssogelos 2017; Diodato and Niglia 2019), or to analyse how these actors have invested foreign policy as terrain for their political practice and agenda once in power (Wojczewski 2019b; Nabers and Stengel 2019; Biegon 2019), but few have investigated how foreign policy outputs have been concretely affected in various national contexts. For instance, the collective volume edited by Frank Stengel, David MacDonald and Dirk Nabers is close to the approach adopted here for its comparative and interdisciplinary angle, but the authors stop short of advancing systematic hypotheses or generalizations as they consider that “differences between various forms of populism will likely also manifest themselves in different foreign policy positions” (Stengel et al. 2019, 6). Third, most of the scholarship on the foreign policy of governing populists has focussed on Latin America (Sagarzazu and Thies 2019; Wehner and Thies 2020; Wajner 2021), India (Plagemann and Destradi 2019; Wojczewski 2019a), Turkey (Taş 2020) and, especially, the US (Drezner 2019; Wojczewski 2019b; Hall 2021). Recent work on Europe has shed light on the foreign preferences of European populist parties (Liang 2007; Balfour et al. 2016; Heinisch et al. 2018; Coticchia and Vignoli 2020; Henke and Maher 2021) and how they have been projected at the EU level (Van Berlo and Natorski 2019; Futák‐Campbell and Schwieter 2020; Falkner and Plattner 2020) as well as on populist parties’ sovereignism more generally (see Basile and Mazzoleni 2020). By contrast, there has been little extensive analysis of the national foreign policies of European populist governments and how they have brought about changes of directions across policy areas (or not). In particular, we lack systematic *comparative* insights on populist governments’ foreign policies in Europe, while some comparative analyses have been undertaken in and across other regions (Destradi and Plagemann 2019; Stengel et al. 2019). This is somehow paradoxical as early research on populist foreign policy preferences was clearly EU-centric.

The special issue aims to address these research gaps by analysing the impact of populism on foreign policy in a range of European cases. By developing a common analytical framework and applying it across several case studies and policy areas, the special issue makes a contribution to nascent efforts at theorizing the relationship between populism and foreign policy, while at the same time providing empirical insights on a number of European cases. Rather than just on populist actors’ preferences in, or utilization of, foreign policy, we aim to shed light on how populism influences foreign policy outputs and processes.

The main research questions guiding our analysis are the following: how does populism impact foreign policy outputs and foreign policy making processes? What is distinctive in the foreign policies of populist governments? Do populist governments bring about change in their country’s foreign policy when they are in office? If so, of what kind and under which conditions?

In order to pave the way for the case-study analyses addressing these questions empirically, this introduction begins with discussing different conceptualizations of populism, then reviews existing studies on its international dimensions, and finally develops theoretical expectations about the impact of populism on foreign policy, which will constitute as many research tracks for the individual contributions. Initial conceptual ground-clearing is necessary for populism remains an essentially contested concept and, more acutely, as developing a common analytical framework allows to maintain coherence across the Special Issue while studying different countries, time periods and foreign policy areas. At the same time, our approach to the concept of populism is explicitly pluralistic since we believe that the diverse understandings of populism developed in the field of Comparative Politics can fruitfully complement each other and help us identify different manners in which populism might influence foreign policy and international politics. In addition, each approach offers various and distinctive opportunities to engage with a number of concepts, theories, or analytical objects—such as securitization, post-structuralism, or diplomacy—that are associated to the IR and FPA scholarship. This mix of coherence and flexibility allows us to gain comparative insights, even though our in-depth case studies are not conceptualized as being part of a systematic small-N comparative research design. Given that the field is new and clearly under-theorized, we do not purport to advance a rigid and comprehensive set of hypotheses about the impact of populism on foreign policy that would invariably apply across all European cases and beyond. Overall, we do not expect populism to translate into a range of uniform and monolithic foreign policy orientations in Europe. Rather, based on the conceptual literature on populism and on the available scholarship on its international implications, we set forth a number of theoretical expectations and identify corresponding research tracks about some of the pathways and modalities by which populism might influence foreign policy. These expectations and research tracks pertain to: (a) the amenability to compromise; (b) bilateralism, multilateralism and support for the EU; (c) the diversification of foreign relationships and international partnerships; (d) foreign policy making processes and diplomatic practice. The different contributions place varying emphasis on these research tracks depending on their relevance in the national context studied.

Each country case-study analyses populist governments’ foreign policies in their specific national, historical and geopolitical context, with a view to determine where, how and when populism might have had an influence. The different countries analysed—namely Austria, Greece, Hungary, Italy, and Poland—have been chosen as they constitute prominent European examples of governing populists. More importantly, they allow for variations across cases. This is true in terms of forms of populist participation in governments (junior coalition partners in Austria and Greece; senior coalition partner in Poland; all-populist coalition in Italy; constitutional majority in Hungary), of ideological orientations (right-wing populism in Austria, Hungary, Italy and Poland; left-wing populism in Greece; sui generis populism in Italy), of time spent in office (e.g. from more of a decade for Hungary’s Viktor Orbán to a little over a year for Italy’s ‘Yellow-Green’ government), and of geographic location (Central, Southern, and Western Europe). More generally, a focus on Europe allows for a rich set of cases of populist governments of different political stripes that are nevertheless embedded in a similar supranational institutional setting. The populist actors studied have not necessarily been in power in their respective countries at the exact same time—some are still in office, others are not—but the decade of the 2010s provides a common periodization for the collective inquiry. Finally, while in this Special Issue the focus is placed on populism in power as a relatively new, potentially consequential and largely understudied phenomenon, this does not rule out the possibility that populist parties might also have an influence on policy-making from the opposition, which has received attention elsewhere (Schain 2006; Balfour et al. 2016; Williams 2018).

## A pluralistic approach to populism

The study of populism has a long tradition in the field of Comparative Politics. In this special issue, we adopt a pluralistic approach to populism since complementary conceptualizations can help us gain insights about different ways in which populism might impact foreign policy.

One of the currently most widespread understandings of populism considers it a ‘thin-centered ideology’. This approach conceptualises populism as a coherent but narrow set of ideas or beliefs that typically coexists alongside a full-fledged ‘thick-ideology’, like socialism or ethno-nationalism (Hawkins et al. 2018). More specifically, in his canonical definition, Cas Mudde (2004: 543, emphasis removed) describes populism as ‘an ideology that considers society to be ultimately separated into two homogeneous and antagonistic groups, “the pure people” versus “the corrupt elite”, and which argues that politics should be an expression of the *volonté générale* (general will) of the people’. Such an approach allows us to capture the commonalities of populist movements and leaders across the political spectrum, from the far-left to the far-right and including those that lack a clear ideological orientation, such Italy’s Five Star Movement or Czech Republic’s ANO (these parties are sometimes associated to ‘pure’ or ‘entrepreneurial’ populism) (Mudde 2010; Zielonka and Rupnik 2020). According to such understanding, populism has two polar opposites: elitism and pluralism (ibid.; Müller 2016). Correspondingly, it entails two constitutive ideological dimensions, anti-elitism and anti-pluralism, both of which might be consequential for foreign policy.

*Anti-elitism* is constitutive to populism as populist leaders characteristically claim to speak in the name of a ‘morally pure and fully unified’ people (*ibid*: 19) as opposed to a predatory class detached from it. Populist discourses’ highly moralistic depictions imply a Manichean worldview in which the ‘people’ is good and elites are ‘evil’ (Mudde 2004: 543; Hawkins 2009: 1043–1044). Depending on the thick ideology that populists espouse, but also on the political context and related opportunities, what counts as predatory elite might vary significantly. Trump attacks the Washington establishment (‘drain the swamp’), Euro-populists focus on EU bureaucracy, and Latin American leftist populists aim at transnational capital and particularly at US businesses and their domestic affiliates.

*Anti-pluralism*, the second core feature of populism, derives from populist leaders’ claim that ‘they, and they alone, represent the people’ (Müller 2016: 3). Depending on the thick ideology, depictions of the people vary and are often kept vague so as to allow for different understanding and thus maximize appeal. Yet, they routinely involve the ‘foregrounding [of] moral distinctions between groups’ (Bonikowski 2016: 22) and the exclusion of certain parts of society, often ethnic or religious minorities. More precisely, right-wing populists generally have an ‘exclusionary’ conception of the people while that of left-wing populists tend to be more ‘inclusionary’ (Mudde and Kaltwasser 2013). Thus, populists’ anti-pluralism does not necessarily coincide with nativism: left-wing populists typically oppose ethno-nationalism. What they have in common, though, is the claim that only they can represent the ‘true people’ and that this excludes political competitors and their constituencies. This is reflected in populists’ characteristic disdain for checks and balances, minority rights (ibid.: 31–32), or ‘any constraint on executive power’ (Drezner 2019: 728). Populists undermine political institutions (Bonikowski 2016: 22; Pappas 2019) by portraying parliaments, courts, the media, and civil society activists as elitist instruments for the control or abuse of the true people (Müller 2016: 31).

A discursive approach to populism, as it was developed by Ernesto Laclau, understands populism not as a set of ideas, but as a ‘logic of political articulation’: “populism does not define the actual politics of these organisations, but is a way of articulating their themes—whatever those themes may be” (Laclau 2005b: 44). According to Laclau, the discursive logic of populism rests on the construction of a chain of equivalence between unsatisfied social demands and the creation of an internal frontier dichotomizing the social into two camps, the power and the underdog (Laclau 2005a, 2005b). By representing the people as a coherent totality and by opposing it to the power or establishment, the populist discursive practice contributes to the very *constitution* of popular subjectivity and to the construction of the identity of the people. Bottom-up movements such as the Yellow Vest in France exemplify well this logic: a popular subject emerged from the aggregation of unrelated social demands on the basis that they are frustrated and that the same source of social negativity (namely President Macron and the establishment) is identified as being responsible. But so does the mobilization of populist discursive strategies by parties such as Syriza (Stavrakakis and Katsambekis 2014) or leaders such as Trump (Homolar and Scholz 2019), who have structured their political rhetoric around antagonistic representations of us-the people versus them-the establishment, with implications for foreign policy.

The discursive approach, therefore, adds to our understanding of populism by shedding light on how populist actors contribute to define (or discursively construct) the social categories they claim to represent (De Cleen and Stavrakakis 2017, 305). In that sense, this understanding of populism allows to account for the diversity and versatility of populist actors and organizations, but also for the dynamic and productive (or performative) nature of populist discourse. As the construction of a chain of equivalence between heterogeneous social demands necessarily implies to “reduce their particularistic content to a minimum”, populists rely on empty signifiers (such as the ‘people’) that are always “open to contestation and redefinition” (Laclau 2005b: 40–41). In ‘filling’ these empty signifiers with meaning, engaging in such contestations or redefinitions, or more generally in promoting certain representations of Self and Other, the populist discursive practice contributes to shape the structure of signification in which politics is debated and policies are formulated (see for instance: Wodak 2015).

Another approach defines populism as a political style, that is as a “repertoire of embodied, symbolically mediated performance (…) that are used to create and navigate the fields of power” (Moffitt 2017: 46). Based on the empirical observation of various populist parties and actors across the world, Benjamin Moffitt (2017; see also: Moffitt and Tormey 2014) inductively identified the following defining features of the populist style: an appeal to ‘the people’ as both the audience and the subject embodied; a resort to ‘bad manners’ and coarsened political rhetoric; and a representation and performance of crisis, breakdown, and threat. The stylistic approach is close to Pierre Ostiguy’s (2017) socio-cultural understanding of populism. According to Ostiguy, political appeals can be meaningfully differentiated as ‘high’ or ‘low’, with populism decidedly and consistently opting for the latter. Populists’ ‘flaunting of the low’ celebrates the concrete, personalized, particularistic, and informal rather than the abstract, impersonal, universal, and formal. Populism in this understanding politicizes socio-cultural differences—‘publicized tastes, language, and modes of public behaviour’ (ibid. 80), as illustrated in official pictures of Donald Trump offering absurd quantities of American fast food at a White House reception or Matteo Salvini’s shirtless posing on Italian beaches. Like the other two, the stylistic approach has the advantage of including both left- and right-wing populists as well as populists parties or movement with less clear political orientation. It also facilitates capturing interesting aspects of contemporary populist leaders’ political communication with important repercussions for international affairs—as, for instance, the public use of undiplomatic language, the employment of social media for foreign policy communications, or the emphasis on personal bonds between world leaders.

Finally, a last conceptual approach understands populism as a *political strategy*, that is, as a set of methods or instruments mobilized by actors in their endeavour to conquer or retain political power. In this understanding, populism is a way to pursue, rather than a ground to define, political goals. According to Kurt Weyland, political strategies are defined by the manner in which they build political support and structure political participation: in that sense, populism is characterized by the reliance on personalized power and “direct, unmediated, uninstitutionalized support from large numbers of mostly unorganized followers” (Weyland 2001: 14). Robert Barr (Barr 2009: 44) expresses the same idea and defines populism as a “mass movement led by an outsider or maverick seeking to gain or maintain power by using anti-establishment appeals and plebiscitarian linkages”. The politico-strategic approach allows to account for the diversity of populist movements, but also for their opportunistic and often erratic political positioning (Weyland 2017). It has mainly been developed with reference to Latin America, but would gain from being applied to Europe as well to better understand how populist actors use foreign policy in the context of their domestic political strategies.

## Populism and Foreign Policy: state of the art

While international factors have been taken into consideration as an explanation for the *emergence* of populism (Zürn 2004; Verbeek and Zaslove 2017; Hooghe and Marks 2018), the literature that specifically assesses the *consequences* of populism for international affairs is scant. Recent analyses have started addressing the impact of populism on specific issues, from their skepticism of international courts and multilateralism (Voeten 2020, 2021) to their approach to international cooperation in the COVID-19 pandemic (Bobba and Hubé 2021). Henke and Maher (2021) have compared the positions of European populist parties on defence policy. Moreover, as emphasised above, some studies have provided valuable empirical insights on the foreign policy preferences of populist actors, but theoretically systematic, methodologically comparative and conceptually pluralistic investigations of the influences of populism on foreign policy are still lacking.

Yet, the literature is growing rapidly, particularly studies grounded in the ideational and discursive approaches to populism. One of the few analyses that assessed the impact of populist government participation on foreign policy is Verbeek and Zaslove’s (2015) study on Italy. They find that while in government the Northern League did not adopt straightforward positions on foreign policy matters, that these positions were defined by how and whether they could be interpreted as ‘protecting the people’, and that the actual impact of the League on foreign policy decisions was largely mediated by the structure of Italy’s political system and coalition dynamics. In another work, the two authors develop systematic expectations about the foreign policy preferences of populist parties (but not of populists in power) on regional integration, trade and finance, migration, and ‘general attitude’ (understood mainly along a nationalist/isolationist/protectionist vs. cosmopolitan continuum) (Verbeek and Zaslove 2017). Their core argument is that populist parties will not necessarily adopt identical foreign policy positions as their preferences appear above all determined by their ‘thick’ ideologies (ibid: 392). In other words, radical right populist parties will have very different views on international affairs as compared to left-wing populists, for example on topics such as immigration or in terms of their isolationist vs. cosmopolitan attitude. In that sense, applying the ideational approach to study foreign policy preferences often leads to displace the analytical focus and explanation away from populism itself and towards thick ideologies instead. For instance, Mihai Varga and Aron Buzogány (2020) argue that traditional conservatism, much more than populism, explain the foreign policy orientations of the Hungarian and Polish populist governments. Leslie Wehner and Cameron Thies (2020) come to similar conclusions regarding the prevalence of thick ideologies in their comparative analysis of the foreign policies of Menem’s Argentina and Chavez’ Venezuela. Relying on role theory, they show that both (populist) leaders brought about change in their countries’ international role conception, but that these changes were driven by their distinctive thick ideologies and respective adherence to the principles of dependency or autonomy. Populism can constitute a narrative or rhetorical frame used to justify role choices, but it does not translate as such into a uniform type of foreign policies (*ibid*. 3).

In this context, Chryssogelos (2017) calls for a comparative approach to the analysis of populist foreign policy while searching for the elements that ‘are themselves a function of traits of populist ideology tout court’ rather than of parties’ thick ideologies. According to his argument, populists’ domestic anti-elitism can explain their opposition to international elites and particularly to the US; populists’ claim of protecting and representing the ‘people’ can explain their suspicion of international or transnational institutions; and populists’ definition of the ‘people’ can transcend national boundaries. While Chryssogelos uses examples from different world regions in a first attempt to specifically address peculiarities of populist foreign policy, he does not systematically test these propositions.

A first plausibility probe of a set of hypotheses on populist foreign policy for the case of India has revealed that the shift to a populist government had the most immediate impact on the process of foreign policy-making and on the communication of foreign policy (Plagemann and Destradi 2019). By contrast, in the Indian case, populism had only a limited impact on the ‘substance’ of foreign policy: contrary to what the authors expected, under a populist leader India’s engagement in global governance did not diminish, and populism did not translate into a rejection of multilateralism. Building upon these insights, Plagemann and Destradi (2019) argue that other factors mitigate the impact of populism on foreign policy. Based on the analysis of the foreign policies of four countries from the Global South (Venezuela, India, Turkey, and the Philippines), they find that the thick ideologies and the structural position of countries in the international system seem to matter in mediating the impact of populism on foreign policy. Yet, in all cases, populists in power tend to centralize foreign policy decision-making. Moreover, the analysis suggests that populists in power tend to reinforce several pre-existing trends in international affairs—a fragmentation of international alliances in particular. In a comparative analysis of Latin American populists’ foreign policies, Wajner (2021) shows they have tended to resort to transnational forms of mutual legitimation and to reproduce domestic approaches to political communication.

Other recent analyses show that a Laclauian framework can also be fruitfully applied to the study of the international implications of populism and, in particular, of the relationship between populism and foreign policy. With reference to Narenda Modi’s India and Donald Trump’s US, Thorsten Wojczewski documents how populist leaders tend to use foreign policy as a site to construct and reproduce their notions of ‘people’ and ‘elite’ as well as their claim to represent the former (Wojczewski 2019a, b). In the same vein, other authors have shown how Donald Trump’s foreign policy rhetoric was put at the service of his populist agenda and, notably, served the function of creating a sense of crisis, of framing the decline of the US ‘heartland’, and of instilling ontological insecurity among the American public (Biegon 2019; Homolar and Scholz 2019; Hall 2021). This, in turn, has consequences on how populist leaders or governments run foreign policy when they are in office. In the US, “the populist elements in the Trumpian foreign policy [notably] manifest themselves in the contestation of the bipartisan consensus on America’s national interest” (Wojczewski 2019b: 17). In Poland, the historical discourse of the PiS government has reflected and reproduced the party’s populist mode of political articulation, with some effects on the country’s foreign policy and diplomatic relations. The common othering of domestic political elites and historical perpetrators, and the totalization of Poland’s victimhood, have connoted the representation of foreign policy situations, of relations with neighbours, and of the state’s identity in international affairs (Cadier and Szulecki 2020). Finally, in her study comparing several cases across regions and time, Erin Jenne has found that populist—and especially ethno-populist—discursive frames correspondingly prescribe diverse forms of foreign policy revisionism (Jenne 2021).

Building upon this literature, we aim to contribute to developing the theorization of populism’s effects on foreign policy by connecting, on the one hand, different approaches to the study of populism in the fields of Comparative Politics and, on the other, the conceptual tools and methods to study foreign policy developed by the disciplines of International Relations and Foreign Policy Analysis.

## Populism and foreign policy: theoretical expectations and research tracks

In this Special Issue, we study in which ways populist government formation or participation has an influence on the foreign policy of a country. More specifically, we analyse the individual foreign policies of populist governments with a view both to identify what might be specific in their orientations, choices and practices as compared to previous non-populist governments and to determine whether and how their coming to—and exercise of—power leads to foreign policy change. In the following sections, we present the research tracks and initial theoretical expectations that have guided our collective inquiry. Our core contention is that populism can be expected to influence a country’s foreign policy, but that it can do so in different ways across cases and even policy areas, and that such impact will be mitigated by other factors.

Analysing the differences with previous non-populist governments and the changes brought upon in a country’s foreign policy allows identifying the potential influence of populism. The FPA literature appears useful in particular to study the modalities, varying degrees and potential sources of foreign policy change (Goldmann 1988; Hermann 1990; Holsti 1982; Welch 2005). Charles Hermann (1990) offers a typology of foreign policy change differentiating between ‘adjustment changes’ (modulation of the same policy), ‘program changes’ (change in methods or means), ‘problem changes’ (change in goals) and ‘international orientation changes’ (fundamental shift in the actor’s role/activities in international politics). When it comes to the drivers of change, the FPA literature identifies a number of potential factors and emphasises their dynamic interactions. For instance, Kjell Goldmann (1988) sets forth a model distinguishing ‘sources of change’ (e.g. changing environmental conditions or learning from previous policies), ‘processes of change’ (e.g. re-thinking by individual leaders or changes in the composition of the foreign policy system) and ‘stabilizers’ mediating change (e.g. cognitive, administrative or international). Through its different case studies, the Special Issue sheds light on how populism acts as, or relates to, sources, processes and stabilizers of change. In line with the FPA scholarship, we do not expect populism to act as a single determinant of foreign policy choices or orientations; rather, we study it in interaction with other factors. In particular, the various contributions identify factors that have mitigated, overridden or amplified the influence of populism on foreign policy, such as external structural conditions and geopolitical pressures, domestic institutional and constitutional architectures, and the thick ideologies of the populist parties or leaders studied.

While individual contributions integrate relevant mediating factors, the thrust of this introduction’s conceptual endeavour lies with advancing a number of research tracks and theoretical expectations about where and how populism might influence foreign policy outputs and processes. We do so below by drawing on the different approaches conceptualising populism and on the nascent literature reflecting on its international dimensions. These expectations and research tracks have guided this Special Issue’s collective inquiry and are further illustrated, substantiated and adjusted in the following individual contributions. They address populists’: (a) amenability to compromise; (b) bilateralism, multilateralism and support for the EU; (c) diversification of foreign relationships and international partnerships; (d) foreign policy making processes and diplomatic practice. These four angles are not necessarily exhaustive—there might be other policy areas or modalities whereby populism influences foreign policy—nor equally valid or important across cases. It has thus been left to authors to identify which research tracks are most relevant in the respective national contexts studied and which policy areas have exhibited variance as compared to previous non-populist governments.

### Amenability to compromise

Among foreign policy observers and in the media, one common assumption about populist actors is that they will adopt a less compromising posture in foreign policy as compared to that of non-populist governments and, overall, anecdotal evidence tends to confirm this impression.

In theoretical terms, different approaches to populism would also lead us to expect populists in power to pursue a more confrontational foreign policy as compared to their predecessors. Populists’ Manichean worldview, highlighted by the ideational approach (Hawkins 2009: 1043) will lead them to depict the world in highly moralistic terms, as a battle of good vs. evil, black vs. white. This, together with populists’ claim to be the only possible representatives and defenders of the ‘true people’ (Müller 2016: 3) might make them less amenable to compromise in international disputes. The literature on populism as a political style also highlights that populists will tend to conjure up crises (Moffitt 2017) and employ an antagonistic, rather than consensual discourse (Ostiguy 2017). Similarly, the discursive approach suggests that the populist logic of articulation rests on the permanent discursive construction of an ‘other’ or ‘enemy’, whether internal or external. This is likely to translate into a confrontational rhetoric towards (certain) other international actors and to shape antagonistic representations of identities. Finally, the politico-strategic approach highlights that populists, after forming governments, need to keep mobilizing their followers. International crises may be particularly suitable to generate domestic support, as is highlighted by the literature on the diversionary theory of war and the ‘rally around the flag effect’ (for an overview and a criticism of existing scholarship, see Tir 2010). Indeed, after they have themselves become part of the governing elite, populists need to keep constructing enemies.

Yet, previous research suggests that shifts to populist governments do not automatically lead to foreign policies that are indiscriminately more conflictive or less amenable to compromise. Relying on operational code analysis, Özdamar and Ceydilek (2019) find that while European Populist Radical Right leaders tend to be more conflictual in their worldviews, they tend to be as cooperative as average world leaders when it comes to their ‘instrumental approaches’. Insights from the Global South suggest that populist governments will pursue a more conflict-prone foreign policy only vis-à-vis countries that are directly associated with a particular section of the population that populists exclude from their definition of the ‘true people’ (Destradi and Plagemann 2019). Finally, the picture is mixed as regard populist parties’ attitudes towards the use of force and intervening in other states’ internal affairs. In the realm of defence, their degree of support for military capabilities and solutions appear largely mediated by their thick ideologies and by national strategic cultures: for instance, most populist radical right party support higher defence spending while left-wing populist parties generally adopt pacifist postures, and while most populist parties tend to favour territorial defence, some support external force projection and military interventions against terrorist groups (Falkner and Plattner 2020; Coticchia and Vignoli 2020; Henke and Maher 2021; see also Wagner et al. 2018).

Given these contradictory findings, case studies in the special issue explore whether, in which ways and under what conditions, populist government formation or participation might lead to less cooperative policies.

### Bilateralism, multilateralism and support for the EU and other International Institutions

One of the areas in which populism seems to have the greatest impact on international affairs in recent years is that of multilateral institutionalised cooperation. Brexit and the Euroscepticism of most European populist parties seems to epitomize populist actors’ aversion to international organizations, as do recent examples of populist governments undermining or withdrawing from global multilateral institutions and regimes (such as the Trump administration pull-out from the United Nations Human Rights Council, the World Health Organization and the Paris Climate Agreement.

In theoretical terms, from the perspective of the ideational approach, we could expect populists’ suspicion of international institutions to mirror their hostility to domestic political institutions, which they habitually accuse of hindering their supposedly direct connection to the ‘people’ (Chryssogelos 2018). More generally, as noted by Chryssogelos (2017), populists represent “a reaction to processes of dilution of popular sovereignty”: “‘sovereignty’ is probably the term that most accurately captures the populist logic of international affairs” (see also: Basile and Mazzoleni 2020). Similarly, the discursive and stylistic approach would lead us to expect populist governments to articulate their identity or posture in opposition to the EU as a technocratic ‘establishment’. Finally, from the point of view of the politico-strategic approach, we can expect populists to make use of voters’ suspicion of far-away transnational elites operating within highly formalized institutional settings to gain political support domestically. Thus, overall, we can expect the formation of populist governments to have a detrimental impact on countries’ multilateral engagement.

Yet, also in this field, previous research reveals that the effects of populism are not necessarily clear and straightforward. Verbeek and Zaslove (2017) have shown that some populist actors—those they characterize as ‘populist market liberals’—are actually rather *supportive* of European integration. More generally, it is worth recalling that although Eurosceptic in their political discourse, the populist governments of Hungary and Poland have not come close to putting their country’s EU membership into question. Furthermore, cases from the Global South reveal that populists’ ‘thick ideology’ or their striving for international status gains might lead to a surprising willingness to engage in international and regional institutions: Venezuela’s Chávez promoted his distinct brand of regionalism in South America as a counterweight to the United States, while India’s Modi was not more averse to regional multilateralism than his non-populist predecessor (Plagemann and Destradi 2019). Finally, while populist governments’ sovereignism often leads them to invoke the principle of non-interference in state’s domestic affairs, they do not necessarily uphold it in practice, as testifies by the Hungarian and Polish government’s policies towards Ukraine and Belarus, respectively.

Hence, more research is needed on populist government’s positions in and towards multilateral organisations and regional integration. In this context, the special issue sheds light in particular on populist government’s attitudes towards both, bilateral vs multilateral diplomatic formats and the EU and European integration. In doing so, contributors notably distinguish between shallow criticism of the EU or other multilateral organizations at the rhetorical level and more substantial policy shifts.

### Diversification of foreign relationships

Another issue that seems particularly relevant for the study of populists in power in Europe is their potential alteration and diversification of international partnerships or alliances. Italy’s pan-populist coalition was the first Western European government to formally subscribe to China’s Belt and Road Initiative. Populist governments in Central Europe have denounced the influence of EU institutions and of Germany and have invested instead in closer ties with Russia (for Hungary) or in alternative regional frameworks (for Poland) (Varga and Buzogány 2020). Finally, the Global South populist governments analysed in Destradi and Plagemann (2019) all try to reduce dependence on a single ally and to diversify their international relationships in order to achieve greater room to manoeuvre for their respective countries.

In the West, populists’ diversification of relationships is likely to dovetail with a more general questioning of some core ‘liberal’ principles on the part of populist leaders—from free trade to liberal democracy. More generally, populists desire to break with long-established foreign policy principles or prioritisation of international partners, which were adopted or favoured by the much-despised previous elites. Similarly, the new representations of Self and Other promoted by populist discourse are likely to have repercussions on how the actions, intentions and roles of other actors are perceived and interpreted.

Against this backdrop, the special issue analyses populist governments’ choices and policies in terms of international partnerships and alliances.

### Foreign policy-making: centralization, personalization, and communication

Besides foreign policy outputs, it is also important to analyse continuity and change in the processes and practices of foreign policy making. Ideational anti-elitism as well as discursive and stylistic drives for differentiation are likely to make populists uneasy with conventional foreign policy-making, which has for centuries been the domain of an exceptionally elitist community of unelected bureaucrats (diplomats), surrounded by a strategic community of think tankers, retired officials, academic experts, and often lower-ranking politicians. Furthermore, the literature on populism as a political style also highlights that ‘bad manners’ are typical of populist leaders and, indeed, we have had several instances of populists in power entirely disregarding diplomatic conventions and etiquette (such as Trump, Salvini or Duterte). In addition, populists’ ideational anti-pluralism and political strategy of cultivating an unmediated link with the people will also make them less willing to involve actors such as civil society representatives or foreign policy (or area) experts in a consultative process. Besides centralization, populists’ claim to *personally* embody the ‘popular will’ and to be the only possible representatives of the ‘true people’ also implies a personalization of foreign policy making (Destradi and Plagemann 2019). In sum, we expect personalization and disregard for diplomatic etiquette to be more pronounced in populist governments as compared to non-populist predecessors.

Yet, apart from anecdotal evidence on spectacular cases, continuity and change of foreign policy-making processes is not easy to assess and remains largely unexplored. Empirical research needs—and the special issue sets for itself—to identify shifts in who the most relevant actors involved in foreign policy-making are, what role is played by foreign ministries and to what extent populist governments deviate from traditional diplomatic practice. Relatedly, it is important to look into the extent to which the established foreign policy bureaucracy opposes populists’ reforms and foreign policy ideas and what the consequences of such bureaucratic infighting are. Furthermore, among the most visible changes of the practice of foreign policy by populists, is the way they communicate both with foreign governments and their domestic support base. A more direct and, perhaps, transparent communication of foreign policy may, on the one hand, contribute to its politicization and polarization thereby complicating consensual international agreements. Yet, it may also increase the publics’ awareness of foreign policy issues, with unclear consequences so far.

Importantly, such changes in the processes of foreign policy making can also be expected to have an impact on the substance of foreign policy. This can happen, for example, through the narrowing down of foreign policy to a limited set of issues that are important to populist leaders—to the detriment of a broader set of issue areas traditionally covered by foreign policy bureaucracies. The marginalization of the traditional foreign policy bureaucracy with its experience in and sympathy for the practice of multilateralism may also deprive populist leaders of the expertise necessary for navigating the intricacies of multilateral institutions and silence government-internal support for doing so.

## Structure of the special issue

The rest of the Special Issue explores these research tracks in depth with reference to individual case-studies (Austria, Greece, Hungary, Italy, and Poland). Overall, the various contributions converge in their findings that the foreign policy of populist governments is most distinctive in terms of foreign policy discourse and style. This does not mean, however, that the impact of populism on foreign policy simply amounts to inconsequential rhetoric or mere posturing: on the contrary, the authors shed light on the different ways in which populist actors have contributed to shape policy outputs and processes, although not in a consistent and uniform manner and not to the level of a radical re-orientation of their country's foreign policy. Several of the theoretical expectations presented above find support in the empirical analyses, though not equally so across cases. Geopolitical contexts, domestic institutional conditions, coalition dynamics and, especially, the duration in office have mediated the influence of populism on foreign policy and help account for differences across national situations.

It is in Central Europe, where populist governments have been in power for several years, that the effects on foreign policy are most salient. In their contribution, Peter Visnovitz and Erin Jenne document a number of changes in Hungarian foreign policy under Viktor Orbán, such as a more confrontational rhetorical stance towards NATO and the EU, an investment in bilateral partnerships with China and Russia, a politicization of foreign policy institutions, and a centralisation of foreign policy-making. They argue that populist argumentation has been used to justify and legitimise these orientations and, more generally, foreign policy revisionism. In that sense, Visnovitz and Jenne show that populist rhetoric neither amounts to 'cheap talk' nor functions as way to signal policy intent in line with constituents' preferences, since the Fidesz electorate's views on the EU, NATO, China or Russia had been at odds with that of their government. They also suggest, however, that years of populist political argumentation seem to have progressively altered these popular attitudes (with aversion towards Russia and China being increasingly supplanted by sympathy), thus shedding light on the potential long-term impact of populism. In the same vein, in his study on Poland, David Cadier argues that populism-induced changes under the Law and Justice (PiS) government have pertained more to foreign policy practices than to foreign policy contents. The reliance on populist discourse and style has led the PiS government to promote distinct representations of Self and Other in international affairs and to use diplomacy as a site to perform a rupture with technocratic elites. More specifically, the PiS government’s foreign policy has been characterized by a shift in the discursive representations of the EU (securitization) and of Poland's role in it (de-europeanisation), by a de-prioritisation of the partnership with Germany and an investment in an alternative Central European core, and by the resort to ‘undiplomatic diplomacy’ and marginalization of traditional diplomatic actors. In that sense, Cadier argues that populist representational practices shape the structures of meaning in which foreign policy is formulated, debated and implemented and, as such, enable certain policy choices and actions while disabling others.

The chapters analyzing cases in Southern Europe observe similar patterns but lower magnitudes of populism’s influence on foreign policy. In his analysis on Greece, Angelos Chryssogelos shows that the effects of populism on foreign policy have been limited, pertaining above all to rhetoric and symbolic actions visible mainly during populist actors’ first six months in office. His cross-temporal comparison of Andreas Papandreou in the 1980s and the SYRIZA-ANEL coalition in 2015–2019 reveals that the former’s maverick foreign policy in the bipolar Cold War context and the latter’s near-rupture with the EU upon entering office were used as a domestic strategy of popular mobilization but that both were eventually abandoned in favour of re-joining the Western mainstream. Chryssogelos thus argues, based on the case of Greece, that populism’s distinctive impact on foreign policy has to do with the ‘how’ rather than with the ‘what’. Populist actors use foreign policy to embody and reproduce the antagonistic relationship between ‘people’ and ‘elites’ as well as the link between the ‘people’ and the leader. However, populism does not constitute a major source of foreign policy change and mainly accentuates pre-existing trends, notably in terms of personalization and centralisation of foreign policy making. This latter, process-related dimension is also well documented in Fabrizio Coticchia’s analysis on Italy: in the all-populist ‘Yellow-Green’ coalition government (2018–2019), Matteo Salvini and Luigi Di Maio constantly and ostentatiously intervened in foreign policy domains that were *not* linked to their respective ministerial portfolios, while the role and influence of the Foreign Minister and Ministry were downgraded. Another feature of the ‘Yellow-Green’ government has been the ideological embracement of sovereignism and a confrontational attitude towards the EU and other multilateral institutions. Yet, Coticchia demonstrates that these positions have mainly been confined to symbolic posturing and have not translated into changes of directions in Italian foreign policy, because populist leaders’ appeared mainly driven by instrumental calculations related to their domestic public image and because institutional and structural constraints (both internal and external) prevented such changes. He suggests though that Italy’s image in the EU has been affected in the process, thus shedding additional light on how, even when mainly confined to rhetoric and posturing, populism may come along with substantial reputational costs amongst other implications for foreign policy.

The last two articles of the Special Issue zoom in on policy domains, issues and processes where the impact of populism is expected—and indeed confirms to be—most salient. Patrick Müller and Charlott Gebauer focus on migration, a policy area where populist parties generally have strong preferences. Analyzing Austria’s policy decision on the UN’s Global Compact on Migration (GCM) they document a clear pattern of change. Whereas the Austrian diplomacy had played a prominent role in the negotiations leading to the GCM, the coalition government made up of the conservative Austrian people’s party (ÖVP) and the right-wing populist Austrian Freedom Party (FPÖ) abstained in the UN General Assembly vote on the pact in December 2018. Müller and Gebauer argue that the populist securitization of the migration issue has led to this outcome. These securitizing practices contribute in turn to shape public perceptions and opportunity structures for other (mainstream) political parties with regard to various policy options, as demonstrated by the fact that the ÖVP not only accepted and adopted the FPÖ’s positions on the GCM but also maintained a critical stance *after* the end of their coalition agreement. Finally, Christian Lequesne picks up and unpacks another common trend in populist governments’ approaches to foreign policy making, namely their attempt to marginalize professional diplomats and Ministries of Foreign Affairs. Relying on a comparison of the cases of Poland, Italy and Austria, Lequesne argues that populist actors are structurally at odds with professional diplomats and seek to reduce their autonomy through political capture. Their success in doing so depends on the institutional conditions prevailing in each national context. In Poland, the PiS government took a series of measures—from recalling Ambassadors to reforming the law on the diplomatic service—that allowed it to politically capture the career diplomatic corps. In Italy and Austria by contrast, the existing legal frameworks, long-established corporatist practices, and political disagreements inherent to coalition politics prevented such outcome. As such, Lequesne’s study sheds light on the need to explore further how populism might affect the relationship between professional politicians and bureaucratic actors.
